# Natural Products as Potential Lead Compounds for Drug Discovery Against SARS-CoV-2

**DOI:** 10.1007/s13659-021-00317-w

**Published:** 2021-09-13

**Authors:** Oyere Tanyi Ebob, Smith B. Babiaka, Fidele Ntie-Kang

**Affiliations:** 1 Department of Chemistry, Faculty of Science, University of Buea, P.O. Box 63, Buea, Cameroon; 2 Institute for Pharmacy, Martin-Luther-Universität Halle-Wittenberg, Kurt-Mothes-Str. 3, 06120 Halle (Saale), Germany

**Keywords:** Antivirals, Drug discovery, Inhibitors, Lead compounds, Natural products, SARS-CoV-2

## Abstract

For the past 2 years, the coronavirus responsible for the COVID-19 infection has become a world pandemic, ruining the lives and economies of several nations in the world. This has scaled up research on the virus and the resulting infection with the goal of developing new vaccines and therapies. Natural products are known to be a rich source of lead compounds for drug discovery, including against infectious diseases caused by microbes (viruses, bacteria and fungi). In this review article, we conducted a literature survey aimed at identifying natural products with inhibitory concentrations against the coronaviruses or their target proteins, which lie below 10 µM. This led to the identification of 42 compounds belonging to the alkaloid, flavonoid, terpenoid, phenolic, xanthone and saponin classes. The cut off concentration of 10 µM was to limit the study to the most potent chemical entities, which could be developed into therapies against the viral infection to make a contribution towards limiting the spread of the disease.

## Introduction

The novel coronavirus disease 2019 (COVID-19) has led to a sudden change in the lifestyle of humans all over the whole world [1–4]. The World Health Organization (WHO) declared the disease resulting from the virus to be a pandemic, which has prompted an exponential increase in scientific research towards finding a drug or a vaccine to limit its spread and the number of casualties. At the time of writing this manuscript, the entire world had experienced about 150 million new infections, about 120 million recoveries and almost 3 million deaths [5]. Two earlier coronaviruses were associated with severe acute respiratory syndrome (SARS) and Middle East respiratory syndrome (MERS). The symptoms associated with COVID-19 include (as in the earlier respiratory syndromes; SARS and MERS) fever, cough, dizziness, and shortness of breath, which might lead to pneumonia and acute respiratory distress, causing death [6, 7]. The virus associated with SARS, MERS and COVID-19 are air-borne and transmissible by contact with infected persons. However, COVID-19 has surpassed the earlier two syndromes in terms of the number of individuals infected and the number of deaths [6].

Coronaviruses (CoVs) belong to the family Coronaviridae, sub-family Coronaviridae and order Nidovirales. Viruses belonging to this family and sub-family are known to be large in terms of genome size (26−32 kb) [8], are enveloped, have a single-stranded ribonucleic acid (RNA) and can infect both animals and humans. CoVs can be subdivided into four genera, i.e., alpha (α), beta (β), gamma (γ) and delta (δ) coronaviruses, according to their genotype and serology [9]. Presently all CoVs that can cause infection in humans belong to the first two categories, i.e., the alpha-coronaviruses (α-CoVs) and the beta-coronaviruses (β-CoVs). Examples of α-CoVs include the human coronavirus Netherland63 (HCoV-NL63) and human coronavirus 229E (HCoV-229E), named after a student specimen coded 229E, while examples of β-CoVs include the human coronavirus Organ Culture43 (HCoV-OC43), the human coronavirus Hong Kong University 1 (HCoV-HKU1), SARS-CoV and MERS-CoV [9].

Even though research on the structure and mode of action of the SARS-CoV-2 virus is ongoing, several known antiviral drugs (e.g. lopinavir/ritonavir, darunavir/umifenovir, oseltamivir, favipiravir, remdesivir, etc.) have been used to manage cases of SARS-CoV infection, together with some anti-malarials like chloroquine and hydroxychloroquine, the antibiotic azithromycin, etc. [10–12]. Besides, many local communities have made claims that some medicinal plants have been successfully used to treat patients who showed some signs of COVID-19 infection [13, 14]. A recent review revealed that 92% of 135 hospitalized patients in northeast Chongqing, China have used traditional medicine in combination with conventional medicine as treatment for COVID-19 [15]. This is because medicinal plants contain natural products (NPs) which hold a huge potential to be used as lead compounds for the development of new drugs [16]. This could include drug leads for treating diseases caused by CoV activity, with the goal of developing them into novel COVID-19 drugs.

Several excellent reviews have recently been published on NPs with the potential for treating SARS-CoV-2 infection [17–19]. Other reviews have focused on antiviral agents with potential for the drug development for the treatment of COVID-19 using crude extracts from plants [20, 21]. However, the focus of such reviews was on the activities of the crude extracts with little emphasis on the isolated bioactive compounds. In some other cases, only in silico hits, i.e. compounds that have been predicted computationally (via molecular docking, molecular dynamics, pharmacophore searches, etc.) have been described [19]. The current study is intended to provide an update of the isolated NPs with the potential to be developed into drugs for the treatment of COVID-19, i.e. compounds showing activities at less than 10 µM against the coronaviruses and/or their target proteins in experimental assays that has been recently published in literature. In some cases, the drug target to which the compounds bind, have been presented in the discussion. The presentation of the compounds is according to the major compound classes, which have been arranged alphabetically.

## Methods

**Fig. 1:**
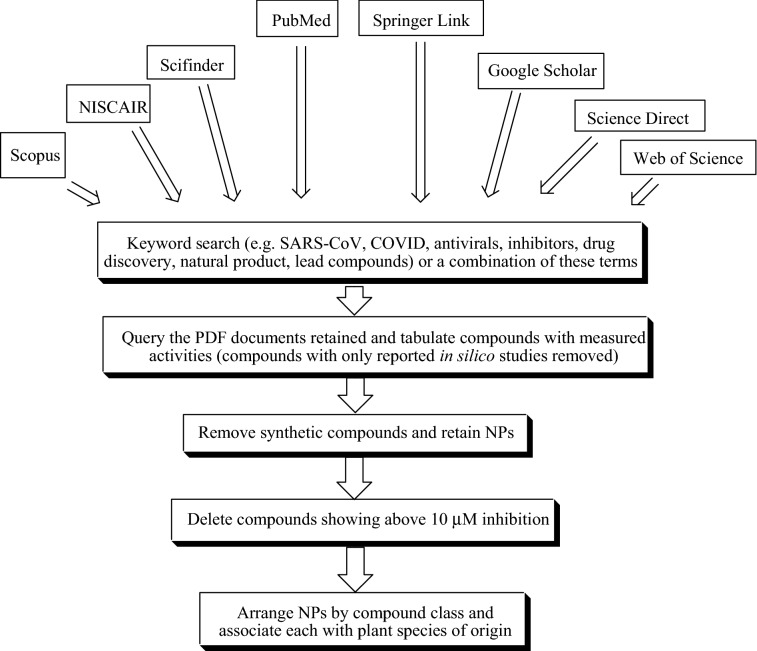
Workflow implemented in this project

All pertinent information about the botanical description, conventional uses, phytochemicals and the pharmacological activities of the isolated compounds were collected from available recently published literature. The electronic databases employed for the assortment of relevant information include Scopus, NISCAIR, Scifinder, PubMed, Springer Link, Science Direct, Google Scholar, Web of Science, and an exhaustive library search. The chemical structures of the compounds were drawn using ChemDraw Ultra 8.0 software. PubChem and ChemSpider databases have been used to check the IUPAC names of the isolated phytoconstituents. The workflow for collecting the literature and writing the report has been provided in Fig. 1.

## Alkaloids

**Fig. 2:**
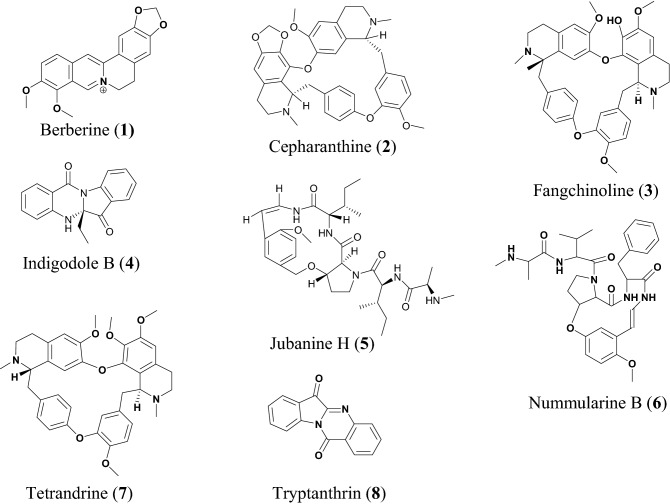
Chemical structures of promising alkaloids (**1** to **8**) with potential against SARS-CoV-2

Alkaloids are nitrogen-containing compounds, mostly known to be bitter principles with diverse biological activities. A summary of alkaloids with potential for drug development against SARS-CoV-2 has been provided in Table 1. Berberine (**1**), Fig. 2, is an isoquinoline alkaloid, known to be biosynthesized by many plant species, particularly those of the genus *Berberis* from which the compound derives its name. Some medicinal properties of **1** reported include anti-inflammatory [40], antiviral [41], antidiabetic [42], antihypertensive [43], hepatoprotective [44] and anticancer [45], additionally is a widely used dietary supplement due to these medicinal properties. According to Warowicka et al. compound **1** could be a possible lead compound against SARS-COV-2, which might act by modulating the nuclear factor kappa of activated B cells (NF-κB) and mitogen-activated protein kinases (MAPKs) [46]. NF-κB is a protein required for DNA transcription and many other cell processes including inflammatory and immune responses. The modulation of NF-κB path is one of the ways by which the alkaloid biberine could inhibit virus infections, in addition to its anti-inflammatory properties [47]. Compound **1** has an inhibitory effect on the NF-κB signaling pathway and therefore might function as an antiviral agent against coronavirus infection. Moreover, during viral infection, the virus regulates expression of inflammatory mediators such as tumor necrosis factor (TNF) [48, 49]. Moreover compound **1** is also known to inhibit herpes simplex virus (HSV) infection both the HSV-1 and HSV-2 virus with EC_50_ values of 6.77 ± 1.13 μM and 5.04 ± 1.07 μM, respectively in a dose dependent manner [50].

**Table 1:** Alkaloids

Compound name	Plant species	Level of activity	Predicted or known target of virus	Mode of action or other activities	References
Berberine (**1**)	*Coptidis* sp.	IC_50_ = 2.0 ± 0.5 μM	MHV-A59		[22]
Cepharanthine (**2**)	*Stephania cepharantha*	IC_50_ = 0.83 ± 0.07 μM	HCoV-OC43		[23]
Fangchinoline (**3)**	*Stephania tetrandra*	IC_50_ = 1.01 ± 0.07 μM	HCoV-OC43	Inhibit viral replication and expression of viral S and N protein	[23]
Indigodole B (**4)**	*Strobilanthes cusia*	IC_50_ = 2.09 μM	HCoV-NL63		[24]
Jubanine H (**5)**	*Zizyphus jujuba*	EC_50_ = 4.49 ± 0.67 μM	PEDV		[17]
Nummularine B (**6)**	*Zizyphus jujuba*	EC_50_ = 6.17 ± 0.50 μM	PEDV, CoV		[17]
Tetrandrine (**7)**	*Stephania tetrandra*	IC_50_ = 0.33 ± 0.33 μM	HCoV-OC43		[23]
Tryptanthrin (**8)**	*Strobilanthes cusia*	IC_50_ = 0.06 μM	HCoV-NL63	Blocking viral RNA genome synthesis and papain-like protease 2 activity	[24]

Cepharanthine (**2**), fangchinoline **(3)** and tetrandrine **(7)** are bis-benzylisoquinoline alkaloids. *Stephania tetrandra* and other species of the genus *Menispermaceae* are major sources of bis-benzylisoquinoline alkaloids which are widely distributed in China and many East Asian countries [51]. A recent review showed the use of *S. tetrandra* in traditional Chinese medicine as antitumour, neuroprotective, anti-inflammatory, and antibacterial [52]. Compound **7** exhibits a wide range of pharmacological activities which include anticancer, anti-inflammatory [51] it is also reported to have inhibitory activity against Ebola virus; herein, tetrandrine acts as a closure of key calcium sensor called two pore channels which remain open during viral infection through endosomal or membrane bound route. [53]. Compound **2** has antiviral activities against human immunodeficiency virus type 1 (HIV-1) virus [54] and HSV-1 virus [55], while compound **3** replication of HIV-1[56]. A time addition assay with MRC-5 cells infected with HCoV-OC43 was used to investigate the antiviral activity of compounds **2**,** 3** and **7**, the compounds strongly inhibited HCoV-OC43 virus with IC_50_ values of 0.83, 1.01 and 0.33 μM, respectively. Additionally these compounds have no cytotoxicity effect on MRC-5 cell at concentrations up to 10 μM [22].

Indigodole B **(4)** and tryptanthrin **(8)** have been isolated from methanol extract of the medicinal herb *Strobilanthes cusia* [57, 58], which is commonly found in northeast India, Bangladesh, southern China, Myanmar, and Taiwan [59]. The roots and leaf extracts of the plant have been used in traditional herbal medicine based on its anti-inflammatory, antimicrobial, and antiviral activities [60, 61]. According to Tsai et al*.* the methanol extract of *S. cusia* reduced HCoV-NL6 viral production with an IC_50_ value of 0.12 μg/mL [24]. Compounds **8** and **4** had high activity against HCoV-NL63, with compound **8** being reported to be the most potent compound, reducing HCoV-NL63 viral production with IC_50_ = 0.06 μM. Meanwhile, compound **4** exhibited an effective virucidal activity against HCoV-NL63 with an IC_50_ of 2.09 μM [24]. Compound **8** specifically altered the antigenic structure of the viral spike protein and further inhibits the cleavage activity of the proteolipid protein 2 (PLP2) gene, associated with virucidal activity, and inhibits the post-entry stage of HCoV‐NL63 replication [24].

Cyclopeptide alkaloids are among the common compounds in plants belonging to the Rhamnaceae, especially *Ziziphus* genus, e.g., *Z. jujuba* [62]. The cyclopeptide alkaloids jubanine H **(5)** and nummularine B **(6)** have been isolated from the stem bark of this species [62, 63]. Compound **6** is also known for its moderate in vitro antimalarial activity (against *Plasmodium falciparum*), with an IC_50_ of 10.3 μM [64]. Compounds **5** and **6** have been tested against porcine epidemic diarrhea virus (PEDV) in Vero cells, with their EC_50_ values established as 4.49 and 6.17 μM, respectively [17].

## Diarylheptanoids

**Fig. 3:**
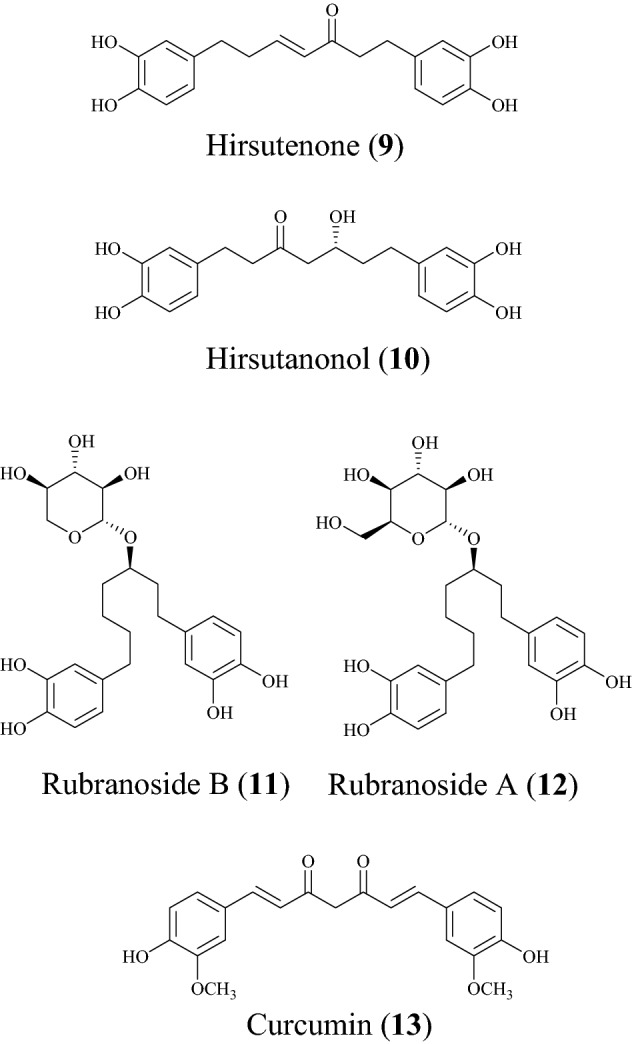
Chemical structures of promising diarylheptanoids (**9** to **13**) with potential against SARS-CoV-2

Diarylheptanoids, also known as diphenylheptanoids, are plant secondary metabolites derived from many plant species. They are made up of phenolic aromatic rings fused together by linear seven carbon chains. They can be either open chain or macrocyclic diarylheptanoids [65]. Diarylheptanoids possess numerous therapeutic benefits including anti-inflammatory [66], antioxidant [67], anti-microbial [68] and anti-diabetic [69] activities. A summary of this class of compounds with potential for drug discovery against SARS-CoV-2 is provided in Table 2, while the chemical structures of the compounds are shown in Fig. 3.

**Table 2:** Diarylheptanoids

Compound name	Plant species	Level of activity	Predicted or known target of virus	Mode of action or other activities	References
Hirsutenone (**9**)	*Alnus japonica*	IC_50_ = 4.1 ± 0.3 μM	SARS-CoV3 PL^pro^		[25]
Hirsutanonol (**10**)	*Alnus japonica*	IC_50_ = 7.8 ± 1.7 μM	SARS-CoV3 PL^pro^		[25]
Rubranoside B (**11**)	*Alnus japonica*	IC_50_ = 8.0 ± 0.2 μM	SARS-CoV3 PL^pro^		[25]
Rubranoside A (**12**)	*Alnus japonica*	IC_50_ = 9.1 ± 1.0 μM	SARS-CoV3 PL^pro^		[25]
Curcumin (**13**)	*Curcuma longa*	IC_50_ = 5.7 μM	SARS-CoV3 PL^pro^		[25]

In a study to discover new compounds that inhibit SARS-CoV, Park et al. found the ethanol extract of the stem back of *Alnus japonica* to exhibit PL^pro^ inhibitory [25]. *A. japonica* has a lot of pharmacologic properties, including anti-inflammatory [70], antioxidant [71] and anti-influenza activities [72]. The isolated diarylheptanoids of interest include hirsutenone (**9**), hirsutanonol (**10**), rubranoside B (**11**) and rubranoside A (**12**) [25]. The isolated diarylheptanoids were tested against SARS-CoV PL^pro^ using a continuous fluorometric assay and showed a dose-dependent inhibitory effect against the PL^pro^ [25]. The compounds were found to be reversible inhibitors because an increase in concentration rapidly reduced enzyme activity. For compounds **9** and **10**, it was found that α,β-unsaturated carbonyl and catechol groups may play a pivotal role in SARS-CoV PL^pro^ inhibition by interacting with the PL^pro^ nucleophiles while the monohydroxy substitution led to drop in the inhibitory effect [73]. When the compounds were tested against SARS-CoV 3CL^pro^, the findings showed that the diarylheptanoids displayed a significant selectivity towards the 3CL^pro^ proteases [25]. The IC_50_ values are 4.1, 7.8, 8.0 and 9.1 µM, respectively. The known viral protease inhibitor curcumin (**13**) was used as a reference inhibitor (with an IC_50_ value of 5.7 µM) [74].

## Flavonoids

**Fig. 4:**
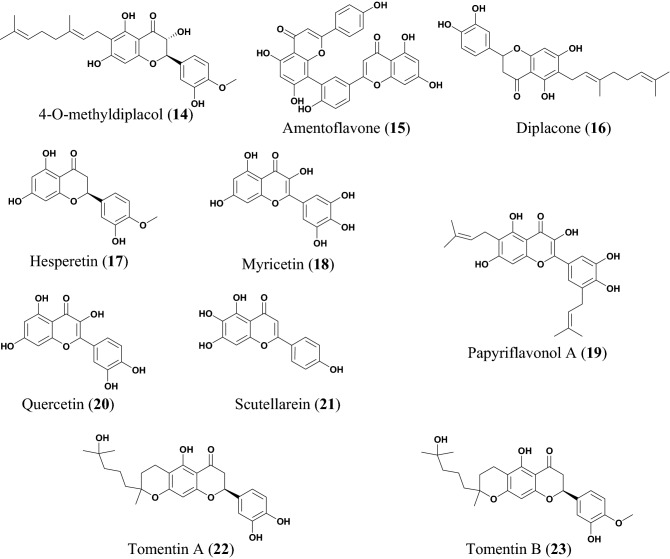
Chemical structures of promising flavonoids (**14** to **23**) with potential against SARS-CoV-2

**Fig. 5:**
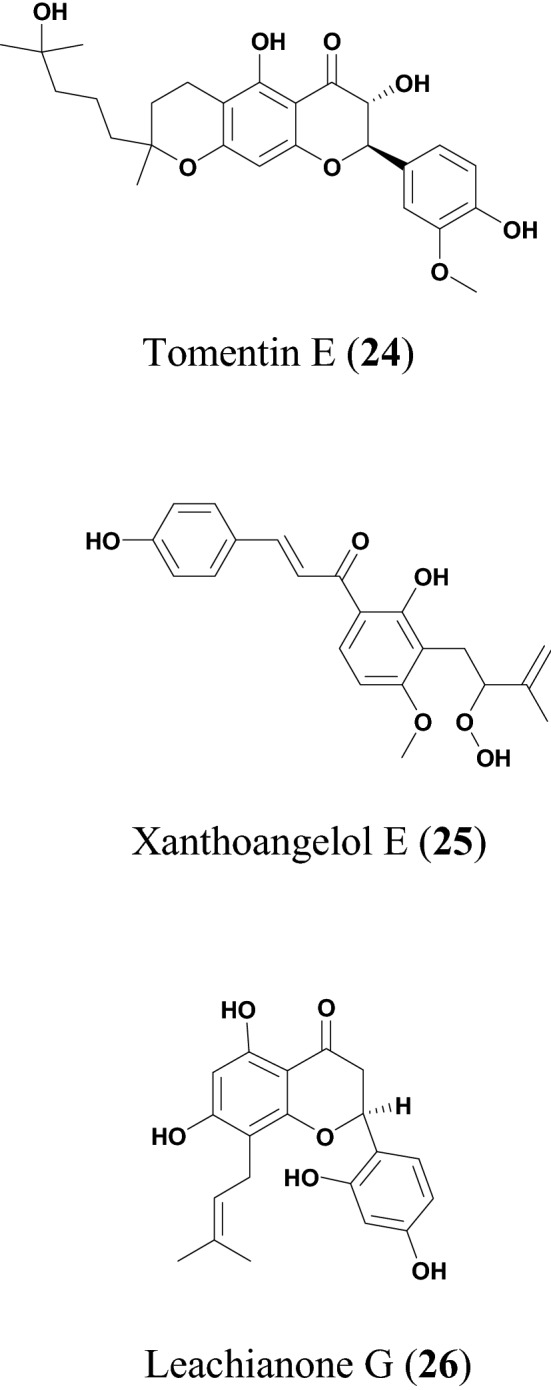
More chemical structures of promising flavonoids (**24** to **26**) with potential against SARS-CoV-2

The potential anti-COVID flavonoids (Figs. 4, 5 and Table 3) include tomentins A (**22**), B (**23**), E (**24**), 4′-O-methyl diplacol (**14**), and diplacone (**16**), which are geranyl flavonoids isolated from the fruits of *Paulownia tomentosa* [26, 27]. *P. tomentosa* is widely distributed in China and parts of this plant species (the bark, fruits, xylem, and leaves) have been used in traditional Chinese medicine (TCM) to treat several ailments, including tonsillitis, bronchitis, asthmatic attacks, and dysentery [75, 76]. The anti-inflammatory property of the plant has been most exploited [77]. Besides, some compounds isolated from the fruits of this species have demonstrated the ability to inhibit airway inflammation [78], while other compounds are known to exhibit cytotoxic [79], antimicrobial and antioxidant activities [80]. The antiviral properties of the fruit extract and the isolated compounds from this species have been investigated in vitro against the polyprotein target papain like protease (PL^pro^), a protein involved in RNA replication [26]. All the compounds from this species displayed a dose dependent inhibition of SARS-CoV PL^pro^, compound **22** being the most potent with an inhibitory constant, K_i_ = 3.5 μM [26]. The IC_50_ values of the compounds are listed in Table 3.

**Table 3:** Flavonoids

Compound name	Plant species	Level of activity	Predicted or known target of virus	Mode of action or other activities	References
4′-O-methyl diplacol (**14)**	*Paulownia tomentosa*	IC_50_ = 9.2 ± 0.13 μM	SARS-CoV		[26]
Amentoflavone (**15)**	*Torreya nucifera*	IC_50_ = 8.3 μM			[27]
Diplacone (**16)**	*Paulownia tomentosa*	IC_50_ = 10.4 ± 0.16 μM	SARS-CoVPL^pro^		[26]
Hesperetin (**17**)	*Isatis indigotica*	IC_50_ = 8.3 μM			[28]
Myricetin (**18**)	*Myrica nagi*	IC_50_ = 2.71 ± 0.19 μM	SARS-CoV NTPase/helicase	Fits in and directly interact with ATP/ADP binding pocket of the SARS-CoV helicase protein, thereby excluding a direct binding of ATP/ADP	[29]
Papyriflavonol A (**19**)	*Broussonetia papyrifera*	IC_50_ = 3.7 ± 1.6 μM	SARS-CoV PL^pro^		[30]
Quercetin (**20**)	*Taxillus chinensis*	IC_50_ = 8.6 ± 3.2 μM	SARS-CoV PL^pro^		[30]
Scutellarein (**21**)	*Scutellarialateriflora*	IC_50_ = 0.86 μM	ATPase activity	Fits in and directly interact with ATP/ADP binding pocket of the SARS-CoV helicase protein, thereby excluding a direct binding of ATP/ADP	[29]
Tomentin A (**22**)	*Paulownia tomentosa*	IC_50_ = 6.2 ± 0.04 μM			[26]
Tomentin B (**23**)	*Paulownia tomentosa*	IC_50_ = 6.1 ± 0.02 μM			[26]
Tomentin E (**24**)	*Paulownia tomentosa*	IC_50_ = 5.0 ± 0.06 μM			[26]
Xanthoangelol E (**25**)	*Angelica keiskei*	IC_50_ = 7.1 ± 0.8 μM	PL^pro^, SARS-CoV 3CL^pro^		[31]
Leachianone G (**26**)	*Morus alba*	IC_50_ = 4.49 μM	HSV-1		[32]

The ethanol extract of *Angelica keiskei* has shown effective inhibition against 3-chymotrypsin-like protease (3CL^pro^) and PL^pro^ (with 75% and 88% inhibition at 30 μg/mL, respectively) [31]. *A. keiskei* is a large perennial plant widely distributed in Japan*,* Korean and China. Its traditional uses include as a tonic, diuretic, laxative, and analeptic [81]. The plant extracts have exhibited several biological activities, including antitumor and antimetastatic effects [82]. The compound xanthoangelol E (**20**) was isolated from *A. keiskei* [31]. Park et al. subjected this compound to fluorescence resonance energy transfer (FRET) and cell-based cis-cleavage inhibition assay to measure the SARS-CoV 3CL^pro^ and SARS-CoV PL^pro^ inhibition in vitro [30]*.* The compound showed a dose-dependent inhibition both in the SARS-CoV PL^pro^ and SARS-CoV 3CL^pro^, with IC_50_ values of 11.4 and 1.2 μM, respectively [30]. These results show that compound **20** has specific inhibitory activity against the cysteine protease.

The crude ethanol extract of *Torreya nucifera* (a plant found in a snowy area near the sea of Jeju island in Korea) was subjected to FRET assay to measure the inhibitory effectiveness against SARS-CoV 3CL^pro^ [83]. The results showed 62% inhibition at 100 μg/mL [27]. The bioflavonoid amentoflavone (**15**) isolated from this species [27], has shown wide pharmacological importance in the treatment of Alzheimer [84] and in the treatment of bladder cancer [77]. Compound **15** also showed potential antiviral activity against the syncytial virus (RSV), with IC_50_ = 5.5 μg/mL [85]. The compound also showed inhibitory effects against the hepatitis C virus (HCC) [86], as well as revealed antiviral activity against influenza-A and influenza-B viruses, although only showing moderate activity against the herpes viruses (HSV-1 and HSV-2) [87, 88]. Moreover, a computational study revealed compound **15** has a good binding affinity against the NS2B–NS3 protease protein in docking simulation (binding affinity of − 9.0 kcal/mol) and showed significant inhibition of the Zika virus from the modelling studies [89]. Ryu et al*.* subjected compound **10** to a fluorescence resonance energy transfer (FRET) assay and showed that this compound displayed a dose-dependent inhibitory activity of SARS-CoV 3CL^pro^ [27].

Myricetin (**18**) is polyhydroxy flavonoid first isolated as a yellow-coloured crystal in the late eighteenth century from the back of *Myrica nagi* harvested from India [90]. The compound is an important element in a variety of human foods, including vegetables, tea and many fruits. Compound **18** is known for its iron-chelating, antioxidant, anti-inflammatory, and anticancer properties [91]. Yu et al. examined the inhibitory effect of compound **18** and scutellarein (**21**) against SARS-CoV helicase, Nsp13, hepatitis C virus (HCV) helicase by using the FRET- bases double stranded DNA unwinding assay as well as using a colorimetry-based ATP hydrolysis assay [29]. Compound **21** was isolated from *Scutellaria baicalensis* (commonly known as Chinese skullcap), which is traditionally used for treating inflammation and respiration [92]. The results showed that compounds **18** and **21** potently inhibited SARS-CoV helicase protein in vitro by acting on ATPase with IC_50_ values of 2.71 and 0.86 μM, respectively, although the compounds did not suppress the helicase activity of the HCV virus [29]. Yu et al*.* modelling analysis revealed that compounds **18** and **21** could fit in and directly interact with ATP/ADP binding pocket of SARS-CoV helicase protein inhibiting the direct binding of ATP/ADP [29]. Moreover, it was observed that compounds **18** and **21** didn’t exhibit cytotoxicity against normal breast epithelial cell lines (MCF10A) [29].

Quercetin (**20**) is a polyphenolic flavonoid found in several vegetables and fruits such as berries, apples, and onions [93]. The compound has been found to have many pharmacological properties, including anti-inflammatory activity by cyclooxygenase inhibition [94–96], lipoxygenase [96, 97], expression of cyclooxygenase and production of prostaglandin E_2_ (PGE_2_), as well as reducing production of interleukin (IL)-1α [98]. The compound also showed antioxidant activity via it's radical scavenging ability [99] and possesses anti-hypertensive activity [100, 101]. Park et al. demonstrated the antiviral activity of compound **20** against SARS-CoV PL^pro^ with an IC_50_ value of 8.6 μM [30]. No cell-based assay of antiviral activity was carried [30]. Besides, compound **20** showed the capacity to block the entry of SARS-CoV into host cells and further antagonized HIV-luc/SARS pseudo typed virus entry with an EC_50_ of 83.4 μM [34]. Additionally, compound **20** showed cytotoxicity, with CC_50_ = 3.32 μM [34].

**Fig. 6:**
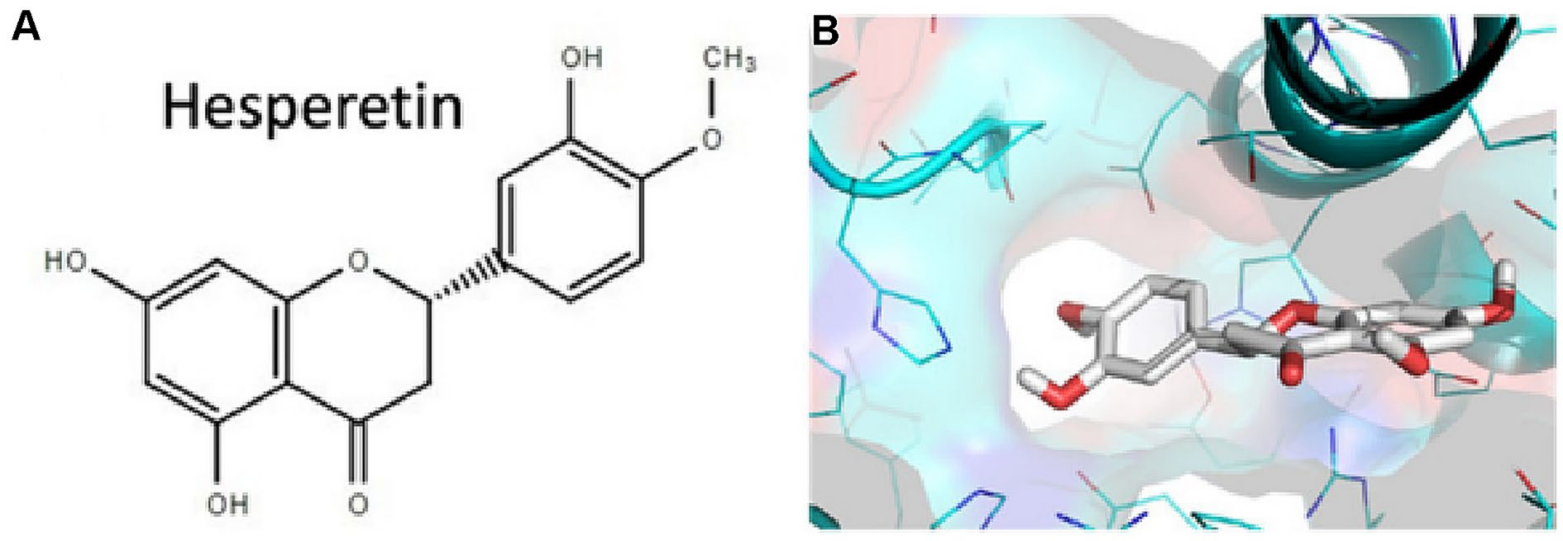
**A** Chemical structure of hesperetin (**17**); **B** The docked pose of the compound towards the SARS-CoV-2 ACE2 receptor binding site for the identification of its putative binding mode. This figure has been reproduced without copyright permission from MDPI because it was originally published under an open access license [104]

Hesperetin (**17**) is a flavone glycoside commonly found in citrus fruits [102]. Recent studies have shown that antioxidant activity of compound **17** is not limited to its radical scavenging effects, but also boosts antioxidant cellular activity through extracellular signal regulated kinases/nuclear erythroid 2-related factor 2 (ERK/Nrf2) signaling pathway [28]. According to Lin et al. compound **17** showed a dose-dependent inhibited cleavage activity of SARS-CoV 3CL^pro^ in cell-based assay [103]. In addition, the compound is less cytotoxic in Vero cells [103]. In silico studies have shown that **17** binds with high affinity to helicase, spike protein and protease side on the ACE2 receptors used by SARS-CoV-2 to cause COVID-19 (Fig. 6), suggesting that this compound could be a potential inhibitor of coronavirus cell growth [104].

Papyriflavonol A (**19**) is a prenylated flavonol from *Broussonetia papyrifera* [105]. *B. papyrifera* is a deciduous tree, whose extracts exhibit antifungal [106], antioxidant [107] and antihepatotoxic properties [108]. The fruits of this plant have been used to treat ophthalmic disorder in China, with its efficacy being proven by pharmacologic experiments [109]. Compound **19** showed a dose-dependent inhibitory effect on SARS-CoV PL^pro^ (IC_50_ = 3.7 μM), when subjected to the fluorogenic peptide Z-RLRGG-AMC assay [30]. The compound also shows a dose-dependent inhibitory effect on both α-glucosidase and cysteine proteases [30]. It is known that PL^pro^ exhibits deubiquitinating (DUB) activity and antagonizes the induction of type-1 interferon (IFN), the interferon-stimulated gene 15 (ISG15) is the most overexpressed gene upon IFN stimulation and it’s involved in marking newly synthesised protein during an antiviral response. Both ubiquitin and ISG15 are important for viral replication and pathogenesis, SARS-CoV PL^pro^ can cleave ubiquitin and ISG15 from cellular conjugates, additionally compound **19** strongly inhibits both ubiquitin and ISG15 with an IC_50_ values of 7.6 and 8.5 µM, respectively [30].

Du et al*.* reported the antiviral activity of leachianone G (**26**) against herpes simplex type 1 virus (HSV-1) [32]. Compound **26** is a prenylated flavonoid present in many plant species particularly *Morus alba* [32]*.* This species is widely distributed in India, China, Japan, and Southern Europe [110]. *M. alba* is also a rich source of phenolic compounds, including flavonoids and anthocyanins which are of great pharmacological and biological importance because of their antioxidant properties [111]. The plant has been used for the treatment of type 2 diabetes mellitus due to its hypoglycemic effects [112]. Moreover, *M. alba* is also known to possess other known antiviral activities, e.g., against rhinovirus [113], dengue virus [114] and hepatitis B virus [115]. The compound showed potent antiviral activity against HSV-1 (IC_50_ = 4.49 µM). Its cytotoxicity tested on Vero cells showed an IC_50_ value of 250 µM. The known antiviral drug (acyclovir), which was used as a positive control in this experiment, also showed potent anti-HSV-1 activity (IC_50_ = 3.65 µM). The antiviral activities and cytotoxic effects of compound **26** as well as for the control antiviral drug (acyclovir) were determined using the viral cytopathic effect assay [32].

## Phenolics

**Fig. 7:**
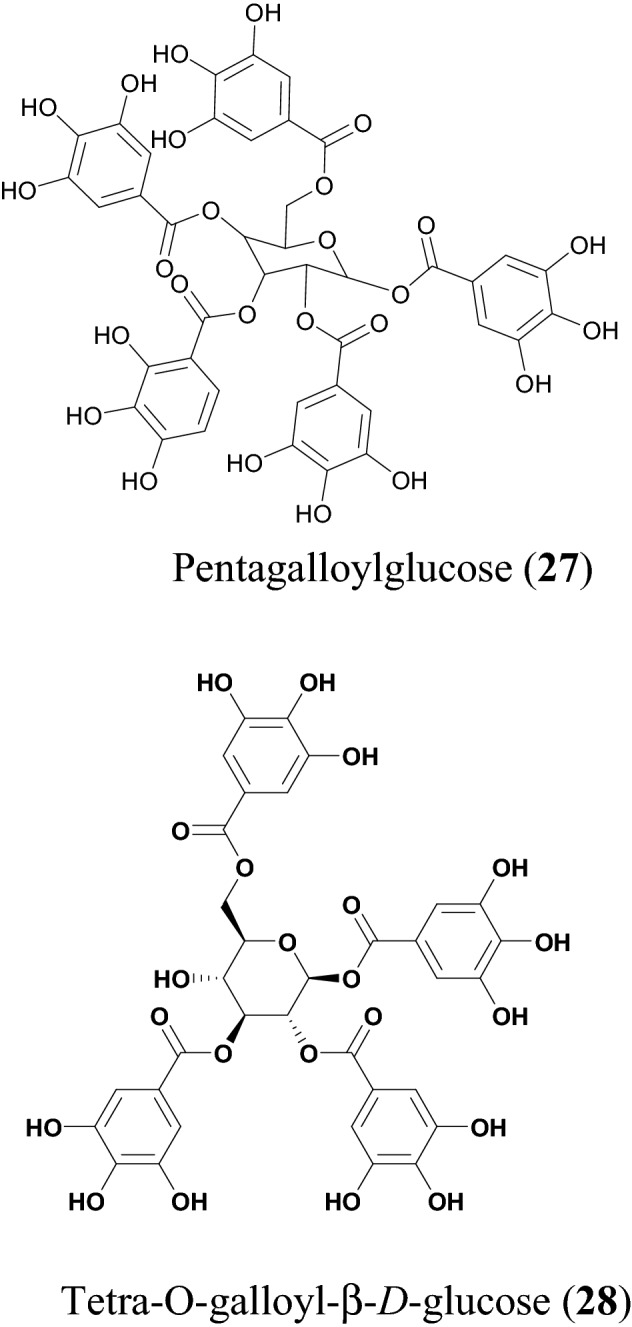
Chemical structures of promising phenolics (**27** and **28**) with potential against SARS-CoV-2

Phenolics are known to be very important dietary components and are strong natural antioxidants [116]. They also exhibit anti-inflammatory, anti-cancer [117], antimicrobial [118] and other pharmacological properties. This class of secondary metabolites contains one or many hydroxyl groups attached directly to a benzene ring, including the flavonoid class earlier discussed [119]. In the paragraphs beneath, we focus the discussion on non-flavonoid-based and non-diarylheptanoid phenolic compounds. A summary has been provided in Table 4, with chemical structures shown in Fig. 7.

**Table 4:** Phenolics

Compound name	Plant species	Level of activity	Predicted or known target of virus	Mode of action or other activities	References
Pentagalloylglucose (**27**)	*Phyllanthus emblica*	EC_50_ = 4.12 ± 0.67 μM	HSV-1	Inhibits HSV-1 DNA and mRNA synthesis (inhibits HSV-1 gene replication, transcription and also block nucleoside nucleocapsid egress and nuclear skeleton rearrangement)	[33]
Tetra-O-galloyl-β-d-glucose (**28**)	*Galla chinensis*	EC_50_ = 4.5 μM	SARS-CoV		[34]

Pentagalloylglucose (**27**) is a polyphenol isolated from the branches and leaves of *Phyllanthus emblica* [120]. It is an indigenous tree in Southeast Asia (also known as Indian Gooseberry or Amla). The extract of *P. emblica* is known to possess great pharmacologic properties, such as anticancer, antitumor and antioxidant activities [121, 122]. Compound **27** has been reported to have several medicinal properties, e.g., as an anticancer agent, since the compound could elicit rapid and selective cytotoxicity in cancer cells [123]. Computational studies reveal that compound **27** could inhibit viral entry by binding to Zika virus envelope protein [124]. Additionally, this compound inhibits the early steps of hepatitis C virus [124], as well as reduces the growth of hepatitis B virus [125] and shows antiviral activities against respiratory syncytial virus [126]. According to Pei et al*.*, the EC_50_ value of compound **27** was measured to be 4.12 µM, using the XTT and plaque reduction assay. Compound **27** shows strong inhibitory activity in early and late stages of HSV-1 virus, inhibiting gene replication, transcription, and related structural changes [33].

Yi et al. investigated the antiviral activity of tetra-O*-*galloyl-beta-d-glucose (**28**) using a colorimetric assay for assessing cell metabolic activity (MTT) assay against SARS-CoV [34]. Compound **28** also expresses an effective SARS-CoV inhibition with an EC_50_ = 4.5 μM and a cytotoxicity value of 1.08 μM using Vero E6 cell following the MTT assay. The results suggest that **28** could be used at concentration to inhibit SARS-CoV without a considerable cytotoxic effect [34].

## Saponin

Saponins are naturally occurring bioorganic compounds having at least one glycosidic linkage (C-O-sugar bond) between an aglycone and a sugar chain. Hydrolysis of a saponin molecule produces two portions, aglycone and a sugar moiety. Specifically, they are naturally occurring glycosides described by the soap-like foaming, and consequently, they produce foams when shaken in aqueous solutions. They are known to exhibit biological properties such as antibacterial, antifungal, antiviral [127] and anti-inflammatory [127, 128].

**Fig. 8:**
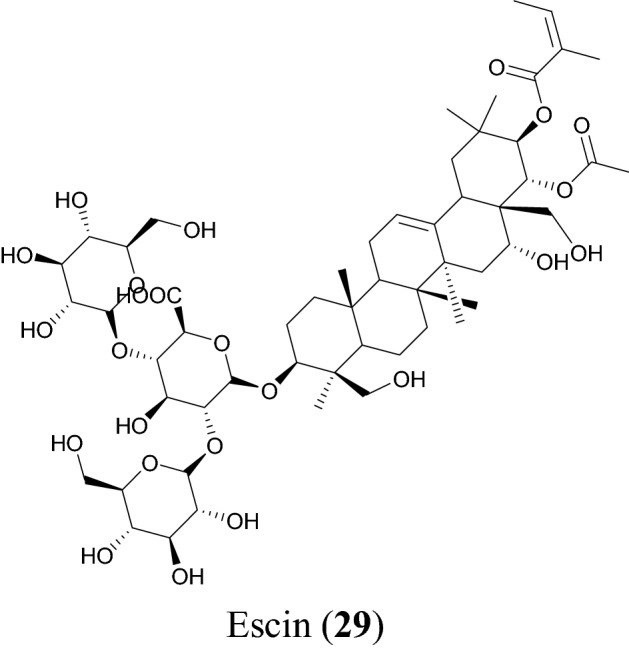
Ecsin (**29**), a promising saponin with potential against SARS-CoV-2

Escin (**29**), Fig. 8 and Table 5, is a major principle from horse chestnut *Aesculus hippocastanum* [129]. The plant has been used in traditional medicine to treat several conditions, including hemorrhoids [130], postoperative edema [131], venomous congestion [132] and anti-inflammatory action [133]. Compound **29** was first isolated in the year 1953 [129] and its pharmacologic and biological properties include anti-inflammatory [134], anti-edematous [132] and preventing the hypoxic damage of the endothelium [135]. According to Wu et al. this compound showed a potent antiviral activity against SARS-CoV-3CL^pro^, its measured EC_50_ value being 6 µM and it had a cytotoxicity value of 15 µM [35]. A cell-based assay with SARS-CoV and Vero E6 cells was used to measure this activity.

**Table 5:** Saponin

Compound name	Plant species	Level of activity	Predicted or known target of virus	Mode of action or other activities	References
Escin (**29**)	*Aesculus turbinata*	EC_50_ = 6 μM	SARS-CoV 3C^pro^		[35]

## Terpenoids

Terpenoids constitute a diverse class of NPs biosynthesized from the condensation of isoprene units to yield terpenes. In plants, terpenoids can serve in communication and defense, for example they act as attractants for pollinators and seed dispersers (chemoattractants or chemorepellents) [115]. Terpenoids are known to possess diverse biological properties, including anti-inflammatory [136], analgesic [137], general antimicrobial [138], and specifically antiviral activities [139]. Potential next generation lead compounds with a premise for SARS-CoV-2 have been summarized in Table 6.

**Table 6:** Terpenoids

Compound name	Plant species	Level of activity	Predicted or known target of virus	Mode of action or other activities	References
*Diterpenoids*					
Dihydrotanshinone I (**30**)	*Salvia miltiorrhiza*	IC_50_ = 4.9 ± 1.2 μM/14.4 ± 0.7 μM	PL^pro^ /CL^pro^	Selective and slow-binding inhibitors for SARS-CoV cysteine proteases	[36]
Tanshinone IIA (**31**)	*Salvia miltiorrhiza*	IC_50_ = 1.6 ± 0.5 μM/89.1 ± 5.2 μM	SARS-CoV PL^pro^ /CL^pro^	Selective and slow-binding inhibitors for SARS-CoV cysteine proteases	[36]
Methyl tanshinonate (**32**)	*Salvia miltiorrhiza*	IC_50_ = 9.2 ± 2.8 μM/21.1 ± 0.8 μM	SARS-CoV PL^pro^ /CL^pro^	Selective and slow-binding inhibitors for SARS-CoV cysteine proteases	[36]
Tanshinone I (**33**)	*Salvia miltiorrhiza*	IC_50_ = 8.8 ± 0.4 μM/38.7 ± 8.2 μM	SARS-CoV PL^pro^ /3CL^pro^	Selective and slow-binding inhibitors for SARS-CoV cysteine proteases	[36]
Cryptotanshinone (**34**)	*Salvia miltiorrhiza*	IC_50_ = 0.8 ± 0.2 μM/226.7 ± 6.2 μM	SARS-CoV PL^pro^/CL^pro^	Selective and slow-binding inhibitors for SARS-CoV cysteine proteases	[36]
*Triterpenoids*					
3β-Hydroxy-28-norolean-12,17-dien-16-one 3-O-6′-methoxy-α-d-glucuronopyranoside (**35**)	*Camellia japonica*	EC_50_ = 0.93 ± 0.22 μM	PEDV		[37]
Celastrol (**36**)	*Tripterygium regeli*	IC_50_ = 10.3 ± 0.2 μM	SARS-CoV 3CL^pro^		[38]
Tingenone (**37**)	*Tripterygium regeli*	IC_50_ = 9.9 ± 0.1 μM	SARS-CoV 3CL^pro^		[38]
3β-Hydroxy-28-noroleana-12,17-Dien-16-one (**38**)	*Camellia japonica*	EC_50_ = 0.28 ± 0.11 μM	PEDV		[37]
3β,16α-Dihydroxy-olean-12-en-28-al 3-O-β-d-glucuronopyranoside (**39**)	*Camellia japonica*	EC_50_ = 0.34 ± 0.01 μM	PEDV		[37]
Pristimerin (**40**)	*Tripterygium regelii*	IC_50_ = 5.5 ± 0.7 μM	SARS-CoV 3CL^pro^		[38]
Iguesterin (**41**)	*Tripterygium regelii*	IC_50_ 2.6 ± 0.3 μM	SARS-CoV 3CL^pro^		[38]

### Diterpenoids

**Fig. 9:**
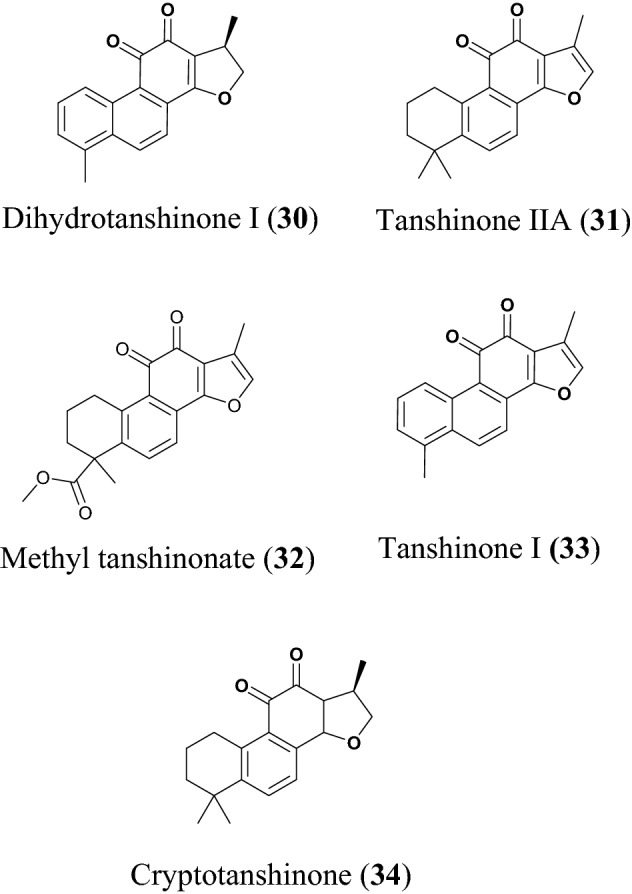
Chemical structures of tashinones, a promising sub-class of diterpenoids (**30** to **34**) with potential against SARS-CoV-2

Park et al. found the ethanol extract of *Salvia miltiorrhiza* to possess great inhibitory activity against both SARS-CoV 3CL^pro^ and PL^pro^. The ethanol extract exhibited 60% and 88% inhibition of 3CL^pro^ and PL^pro^, respectively, at 30 µg/mL). The plant species *S. miltiorrhiza* is widely found in China, Korea, and Japan and has been greatly used to treat coronary heart disease, particularly angina pectoris and myocardial infarction [140]. *S. miltiorrhiza* is known to possess antioxidant, anti-inflammatory and antiviral properties [39]. Tashinones (Fig. 9) isolated from *S. miltiorrhiza* include dihydrotanshinone I (**30**), tanshinone IIA (**31**), methyl tanshinonate (**32**), tanshinone I (**33**) and cryptotanshinone (**34**) [36]. All the tanshinones are good inhibitors of the cysteine protease (3CL^pro^ and PL^pro^), for 3CL^pro^ the inhibitory activity was assayed following the proteolysis of the fluorogenic substrate in the presence or absence of the test compounds, all compounds except compound **33** exhibited a dose dependent inhibitory effect on 3CL^pro^ the activities ranged from 14.4 to 89.1 µM while PL^pro^ inhibitory activity was assayed following continuous fluorometric, these compounds impressively show better activity against PL^pro^ have a time dependent inhibitory profile on PL^pro^ with compound **33** having highest activity ( IC_50_ value of 0.8 µM), all compounds have better activity against PL^pro^ compared to 3CL^pro^ [36]. A detailed kinetic mechanism study showed that compounds **30**, **31**, **32**, **33** and **34** exhibited slow binding inhibitions with enzyme isomerisation and were shown to be non-competitive inhibitors. Moreover, compound **33** was reported to have a potent activity against cellular DUB with IC_50_ = 0.7 µM [36]. Recent studies have reported the use of compound **31** to treat myocardial infarction and delay of ventricular remodeling, in combination with puerarin. The compound is known to act by inhibiting the inflammation in the early stage of myocardial infarction and plays an important role in inhibiting the ventricular remodeling in the later stage of myocardial infarction. The combination of compound **31** and puerarin can improve cardiac function, improve hemodynamics, reduce myocardial cells and reduce collagen synthesis in mice after myocardial infarction [141].

### Triterpenoids

**Fig. 10:**
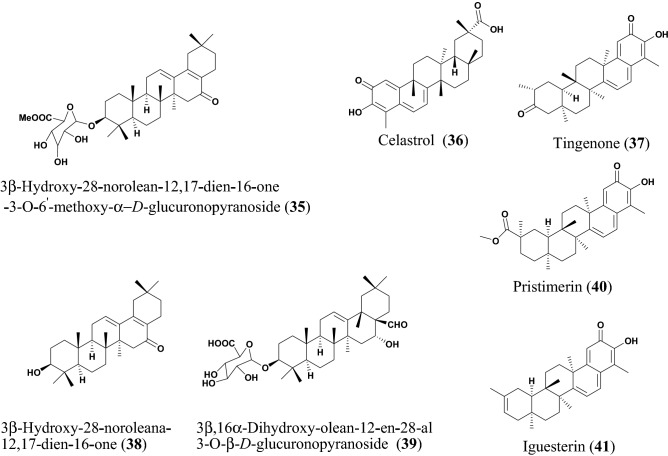
Chemical structures of promising triterpenoids (**35** to **41**) with potential against SARS-CoV-2

Yang et al. isolated: 3β-hydroxy-28-norolean-12,1-dien-16-one 3-O-6′-methoxy-α-d-glucuronopyranoside (**35**), 3β-hydroxy-28-noroleana-12,17-dien-16-One (**38**), and 3β,16α-dihydroxy-olean-12-en-28-al 3-O-β-d-glucopyranoside (**39**), which are all oleanane triterpenes (Fig. 10) from the flowers of *Camellia japonica*. The compounds were tested for their inhibitory effectiveness against PEDV [135]. By targeting the epithelial cells of the small intestine, the PEDV virus can cause severe mucosal atrophy and malabsorption resulting in acute and lethal diarrhea in piglets [127]. The plant species *C. japonica* is found abundantly in Korea, Japan, and China [128]. The ethanol extract (70%) of *C. japonica* has a potential inhibitory effect against PEDV replication [133]. The anti-inflammatory [33] and cytotoxic [116] properties of flowers of *C. japonica* have been exploited traditionally and used in treating hematemesis and internal and external bleeding injury [142]. The compounds **35**, **38** and **39** had potent effects against the replication of PEDV. The known drug azauridine was used as a positive control. Additionally, the compounds gave higher selective indices (SI = 14.74, 32.72 and 6.68, respectively), with compound **38** being lower than azauridine (SI = 14.30). Moreover, compound **38** was shown to inhibit the virus replication in a time course study and it was further investigated detailly and found to inhibit PEDV RNA expression, encoding nucleocapsid, spike and membrane protein in a dose-dependent manner at concentrations of 2.0, 1.0, 0.5, and 0.25 µM, respectively [135]. Therefore, the anti-PEDV molecules **35**, **38** and **39** could be investigated further as candidates against SARS-CoV-2.

**Fig. 11:**
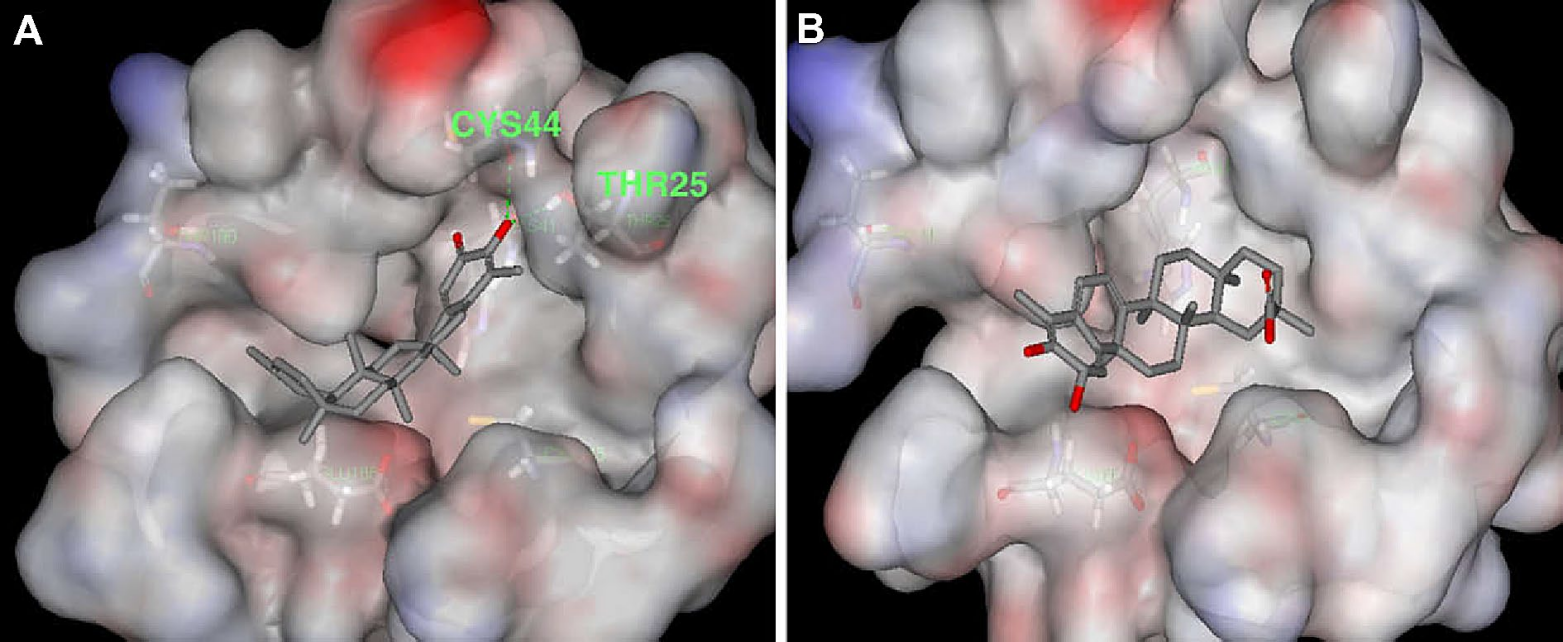
Molecular docking poses of the triterpenoids iguesterin (**A**) and dihydrocelastrol (**B**), isolated from the plant *T. regelii* showing binding to SARS-CoV 3CL^pro^ (PDB code: 1uk4). This figure has been reproduced with copyright permission from Elsevier [38]

Celastrol (**36**), tingenone (**37**) pristimerin (**40**) and iguesterin (**41**) are quinone methide triterpenoids isolated from the bark of *Tripterygium regelii* [143]. *T. regelii* is a vine found widely in China, Korea, Japan, and Taiwan [117]. *T. regelii* plant has a good cytotoxicity property against numerous cancer cell lines [118] and its efficacy has been reported in rodent models of arthritis and other inflammatory disease [119, 120]. Ryu et al. reported the SARS-CoV 3CL^pro^ inhibitory properties of compounds from this plant species [143]. All compounds displayed dose-dependent inhibitory activities, with the compounds **36, 37**, **40** and **41** having IC_50_ values of 10.3, 9.9, 5.5 and 2.6 µM, respectively. Additionally, structure–activity relationship studies revealed that the quinone-methide moiety present in these compounds is important for SARS-CoV 3CL^pro^ inhibition (Fig. 11) [38]. Molecular docking analysis of compounds from this class towards the 3CL^pro^ protein with Protein Data Bank (PDB) code 1uk4 revealed that compound **41** could fit well into the substrate-binding pocket of SARS-CoV 3CL^pro^, with the hydroxyl group of C3 of compound **41** forming a hydrogen bond with the oxygen atom of the carbonyl group of Cys44 and the OH of Thr25 located in domain I of the protein drug target (Fig. 11) [38].

## Xanthone

Xanthones are oxygen containing heterocyclic compounds commonly occurring in higher plants (especially those of the Guttiferae, Gentianaceae, Moraceae, Clusiaceae and Polygalaceae families), fungi, lichen, and bacteria [144, 145], this class of secondary metabolites have been known to contain the following biological properties antimicrobial [146, 147], anticancer [146, 147], anti-inflammatory [146, 147], antimalaria [146, 148] and antiviral [149].

**Fig. 12:**
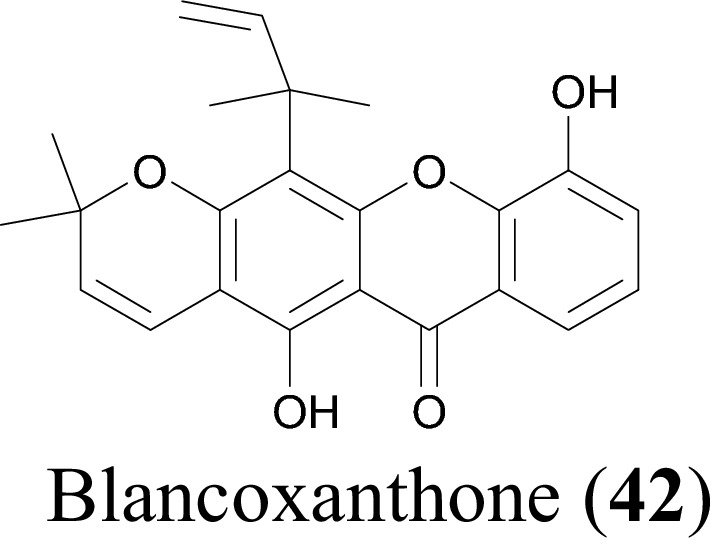
Blacoxanthone (**42**), a promising xanthone with potential against SARS-CoV-2

Shen et al. studied the antiviral activity of *Calophyllum blancoi* against SARS-CoV. The genus *Calophyllum* is a rich source of phenolics and xanthones, possess great antibacterial [143, 150], antifungal [151], antiplatelet aggregation [150], immunomodulatory [152], anticancer [153] and anti-HIV-1 viral activities [154]. Blancoxanthone (**42**), Table 7 and Fig. 12, isolated from the roots *C. blancoi* [42] showed the greatest in vitro inhibitory activity against HCoV-229E with an EC_50_ value of 7.93 µM [42]. The antiviral activity of the compound was evaluated using the Cell Proliferation Kit II (XTT) [42].

**Table 7:** Xanthone

Coumpound name	Plant species	Level of activity	Predicted or known target of virus	Mode of action or other activities	Reference
Blancoxanthone (**42**)	*Calophyllum blancoi*	IC_50_ = 7.93 μM	HCoV-229E		[39]

## Discussion

Out of 42 NPs belonging to the alkaloid, flavonoid, terpenoid, phenolic, xanthone and saponin classes obtained from our literature survey. Our results showed that majority of the NPs with inhibitory concentrations against the virus or its target proteins below 10 µM were terpenoids, alkaloids, flavonoids, and diarylheptanoids, respectively. The terpenoids (diterpenoids and triterpenoids) were the most abundant among the isolated NPs, constituting 26.19% of the isolated compounds. This was followed by alkaloids (19.05%), flavonoids (11.90%), diarylheptanoids (9.52%), phenolics (4.76%), saponin (2.38%) and xanthones (2.38%), which showed a similar trend to previous studies [17–19].

## Conclusion

Natural products (NPs) have provided privileged scaffolds in drug design [16]. The novel coronavirus disease-2019 (COVID-19) is caused by a positive-strand ribonucleic acid (RNA) virus, the severe acute respiratory syndrome coronavirus 2 (SARS-CoV-2) [155]. The virus has infected several million people and caused thousands of deaths worldwide since December 2019 [156]. The pandemic is a significant threat to public health and the global economy [155]. To search for new bioactive compounds with anti-SARS-CoV-2 activity, 42 NPs with inhibitory concentrations against coronaviruses, their target proteins and other viruses, e.g., HIV. Influenza A virus, human simplex virus or their target proteins which lie below 10 µM were identified from published data in literature. The SARS-CoV-2 virus was only discovered in 2019. References that show the testing of NPs against other SARS-CoV viruses (e.g. HCoV-NL63) and their targets (e.g. SARS-CoV PL^pro^, 3CL^pro^, SARS-CoV helicase, etc.) had earlier been published. We are presenting this review to encourage the testing of these compounds against the SARS-CoV-2 virus. In writing this manuscript, we must mention that the compounds were actually tested against the SARS-CoV-2 virus. Only ten of the 165 references date after 2020. It was the goal of the authors to summarize the results of compounds and plant species whose extracts have already been tested against earlier discovered coronaviruses so as to ease the task of discovery of NP antivirals that could be next generation drugs to treat infections caused by SARS-CoV-2. The results of the current study reveal that promising NPs as potential inhibitors of SARS-CoV-2 were terpenoids, alkaloids, flavonoids, and diarylheptanoids, respectively. The mechanism of action of some of the NP hits and the plant species from which they were isolated has been included in the study. It is worth mentioning that some of the medicinal plants and the NPs have shown good safety in vitro studies. This review could serve as a starting point for further development of these NPs hits as potential leads for COVID-19. Thus, it would be interesting in future to evaluate the toxicities and binding mode of some of these NPs using in silico approaches.
